# Normal ranges of tissue Doppler imaging echocardiographic parameters in healthy term and preterm newborns: a systematic review and meta-analysis

**DOI:** 10.1007/s00431-025-06323-1

**Published:** 2025-08-14

**Authors:** Martina Ciarcià, Marco Andrea Malanima, Benjamim Ficial, Enrico Petoello, Alice Iride Flore, Salvatore De Masi, Ersilia Lucenteforte, Carlo Dani, Simone Pratesi, Iuri Corsini

**Affiliations:** 1 Neonatal Intensive Care Unit, University and Hospital Trust of Verona, Verona, Italy; 2 Unit of Medical Statistics, Department of Clinical and Experimental Medicine, University of Pisa, Pisa, Italy; 3 Clinical Trial Center, University Hospital Careggi, Florence, Italy; 4 Department of Statistics, Computer Sciences and Applications “G. Parenti” University of Florence, Florence, Italy; 5 Department of Neurosciences, Drug Research and Child Health, University of Florence, Florence, Italy

**Keywords:** Tissue Doppler imaging, Echocardiography, Neonatology, Normal range TDI

## Abstract

Reference values for tissue Doppler imaging (TDI) parameters in neonates remain limited and are not consistently defined across gestational ages or postnatal periods. We conducted a systematic review and meta-analysis to define normal ranges for TDI-derived myocardial performance indices in healthy term and preterm newborns, stratified by gestational age (GA) and day of life (DOL). We searched MEDLINE, EMBASE, and CENTRAL up to April 2024 for observational studies reporting TDI measures in neonates grouped by GA (< 32, 32–36, ≥ 37 weeks) and DOL (≤ 7, > 7). Study quality was assessed using the checklist, and evidence certainty was evaluated with a modified GRADE approach. Thirty-five studies including 3747 neonates were included in the analysis, comprising 1635 term and 2112 preterm infants. We analyzed peak systolic (s′), early (e′) and atrial (a′) diastolic velocities, as well as isovolumic contraction and relaxation times, ejection time, and ventricular filling time. TDI parameters increased with GA and DOL, reflecting early postnatal hemodynamic changes. *Conclusion*: This meta-analysis provides robust, clinically relevant reference ranges for TDI parameters in neonates across gestational and postnatal ages, supporting their broader use in functional cardiac assessment and informing future research directions.

**Table:** 

**What is Known:** • *Tissue Doppler imaging (TDI) has been widely applied to assess myocardial function in neonates. Reference ranges for TDI parameters in newborns exist but are not uniformly established across gestational ages and postnatal days. Variations in myocardial velocities and cardiac cycle times are known to reflect developmental changes in cardiac function.*	
**What is New:** • *This systematic review and meta-analysis define comprehensive normal ranges for TDI velocities and cardiac cycle time intervals in healthy term and preterm newborns, stratified by gestational age and days of life. It highlights hemodynamic adaptations in early life and provides clinically relevant benchmarks for assessing neonatal cardiac function.*

**Supplementary Information:**

The online version contains supplementary material available at 10.1007/s00431-025-06323-1.

## Introduction

Echocardiography has been increasingly used in neonatal intensive care units (NICUs) to assess the hemodynamic status of critically ill neonates and to support clinical decision making [1]. Recent guidelines by the American Society of Echocardiography, endorsed by the AAP and AHA, emphasize the clinical relevance and standardization of targeted neonatal echocardiography (TnECHO) in the NICU setting [2]. Echocardiographic quantification of myocardial function plays an essential role in the assessment of these patients. Shortening fraction and ejection fraction quantify left ventricular systolic function by measuring changes in dimension and volume respectively. However, these conventional measurements have several shortcomings: poor sensitivity in identifying myocardial dysfunction, suboptimal reproducibility, and reliance on normal left ventricular geometry [2].

New imaging modalities have been applied first in adults and then in neonates to more accurately assess myocardial performance.

**Fig. 1:**
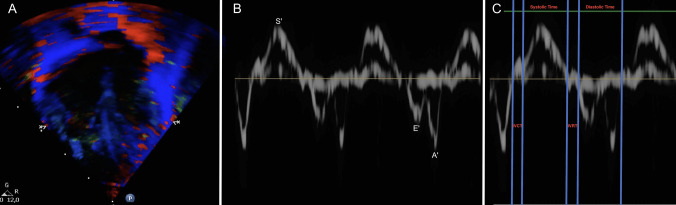
**A** Tissue doppler imaging (TDI) in the apical four-chamber view. Myocardial velocities are conventionally measured at the septal and lateral insertion points of the mitral and tricuspid valve for septal, right and left ventricle, respectively. **B** Measurement of peak systolic velocity (s′) and the two diastolic peak velocities—early diastolic (e′) and late diastolic or atrial (a′) waves—corresponding to passive and active ventricular filling, respectively. **C** Measurement of the time intervals of the four phases of the cardiac cycle: isovolumic contraction time (IVCT), ejection time (ET), isovolumic relaxation time (IVRT), and ventricular filling duration. (FT)

Tissue Doppler imaging (TDI) is a relatively new technique that quantifies myocardial performance by measuring myocardial velocity and cardiac cycle timings. TDI has been widely used in adults and older children to evaluate both systolic and diastolic function of the left and right ventricles [3]. TDI relies on the Doppler principle to calculate the velocity of a specific point of the heart by measuring the frequency shift of ultrasounds reflected by the myocardium. Myocardial velocities are conventionally measured on the septum (IVS) and lateral insertion of the mitral and tricuspid valve for the left (LV) and right ventricle (RV) respectively, from a 4-chamber view (Fig. 1A). The resulting velocity curve (Fig. 1B) allows the measurement of peak systolic velocity (s′) and the two diastolic peak velocities (e′ and a′), corresponding to early and active filling, respectively. TDI also enables the measurement of the four phases of the cardiac cycle (Fig. 1C): isovolumic contraction time (IVCT), ejection time (ET), isovolumic relaxation time (IVRT), and ventricular filling time (FT) [3].

Numerous studies have reported differences in TDI indices between healthy neonates and those with various conditions (e.g., bronchopulmonary dysplasia, perinatal asphyxia, respiratory distress syndrome, patent ductus arteriosus, sepsis, anemia) [4, 5]. Interestingly, these differences were not detected by conventional echocardiographic parameters. TDI indices tend to increase with gestational age (GA) and time, likely reflecting heart size, maturation, and improved function. Although reference ranges for neonates have been proposed, considering GA and day of life (DOL), normal values have not been firmly established.

Current recommendations suggest basing interventions in critically ill neonates on the evaluation of both clinical and echocardiographic parameters rather than relying solely on TDI indices [3]. We believe that establishing normal range values according to GA and DOLis crucial for integrating TDI into routine cardiac function assessment in neonates. Therefore, we conducted a systematic review and meta-analysis of the available literature to define the normal ranges of TDI measures of myocardial performance (s′, e′, a′, IVCT, ET, IVRT, FT) in healthy term and preterm neonates at different GA and DOL.

## Materials and methods

### Study design and search strategy

We conducted this systematic review in accordance with the PRISMA 2020 reporting guidelines [6]. The protocol was registered on PROSPERO (CRD42022297368).

We searched MEDLINE, EMBASE, and CENTRAL databases from inception to April 1 st, 2024, with English language restrictions. Reference lists of included studies were also checked.

Search strategies are available in Supplementary Table 1 (Tab S1).

### Study selection and quality assessment

Titles and abstracts were screened to select eligible studies, followed by full-text evaluation. Two independent investigators (MC, BF) reviewed each study, with discrepancies resolved by a senior supervisor (IC). Two authors (MC and BF) independently extracted data from the included studies. Discrepancies were resolved through discussion with a third reviewer (IC).

We included observational, non-comparative studies of healthy term or preterm newborns grouped by GA: < 32 weeks, 32–36 weeks, and ≥ 37 weeks. Cardiac TDI parameters (left, septal and right) were measured during the first month for term infants or up to 44 weeks for preterms. Healthy infants were defined as those not requiring supplemental oxygen or respiratory support, except for < 32 weeks, where non-invasive support with FiO2 < 0.3 was accepted [7].

We excluded studies with irrelevant topics, non-original studies, and those lacking TDI parameters or clear GA or time of measurements. Study quality was evaluated (MC, IC) using the Joanna Briggs Institute Critical Appraisal Checklist (JBI) for prevalence data [8]. Disagreement between reviewers was resolved through formal discussion. Studies scoring 9 on the JBI checklist were considered high quality.

Data were extracted into a database (Microsoft Excel), including study design, sample characteristics (GA, birthweight, gender, sample size), and TDI measures (s′, e′, a′ velocities, IVCT, IVRT, ET, and FT) for right, left, and septal regions. All studies included in our meta-analysis reported pulsed wave TDI-derived measures.

### Statistical analysis

Meta-analyses were performed using random-effect models. Inverse variance was used for weighting, and restricted maximum likelihood (REML) for estimating between-study variance *τ*^2^. Parameters were analyzed by GA categories and DOL (≤ 7, > 7). Means and standard deviations (SD) were primarily used; if unavailable, medians and IQRs were converted to means and SDs. We assumed the distribution of the outcomes to be similar to normality. In case of missing values of the mean, we replaced them with the values of the median. When the SD was missing, we obtained it by dividing the interquartile range by 1.35 [9].

Heterogeneity was assessed with Cochran’s *Q* test, with significance at *p* < 0.10. Meta-regression analyses were also carried out to assess the association between the mean gestational age and parameters a′, e′, s′. When mean GA was not available, we used the median or, in case neither this value was reported, the maximum value minus the minimum value divided by two. When no information on GA was not available, the corresponding study was excluded from meta-regression analysis. We used random-effect models with REML estimation of *τ*^2^ and Hartung and Knapp method to adjust test statistics and confidence intervals.

### Certainty of evidence

We applied a modified GRADE approach [10] to evaluate evidence certainty, starting from low due to non-randomized designs. As in the traditional GRADE approach, the certainty of the evidence could be downgraded for risk of bias (downgraded one level if serious or unknown risk of bias in one or more domains), inconsistency (downgraded one level if *p*-value for heterogeneity test < 0.10 or only one study was available), indirectness (downgraded one level for indirectness in populations), and imprecision (downgraded one level if for wide confidence interval or total sample size < 50). In accordance with the GRADE approach, the certainty of the evidence from non-randomized studies may be upgraded for a very large estimate of effect. As our study did not estimate effects, we decided to upgrade the certainty of the evidence one level if evidence came from study with large sample size (total sample size > 250).

## Results

### Study selection

**Fig. 2:**
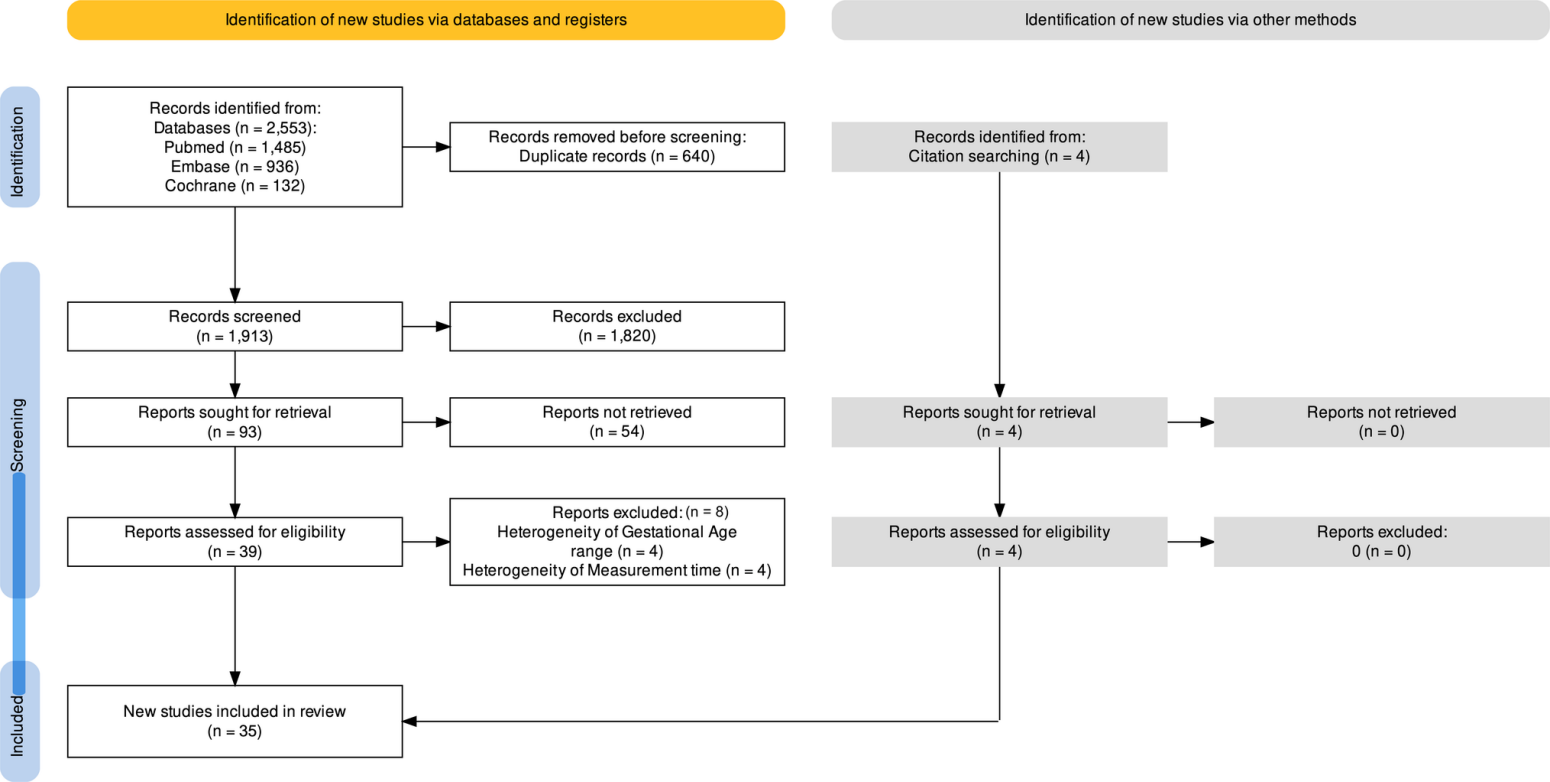
PRISMA flow chart of the study selection

Figure 2 shows the PRISMA flow chart of the study selection. Our search strategy identified 1904 articles, after duplicate removal. Following abstracts and titles screening, 84 studies met the inclusion criteria. After full-text analyses, 35 studies [7, 11–45] were eligible and were included in the meta-analysis.

Sixteen papers reported data about TDI parameters of right ventricular function, 21 about the lateral wall of the left ventricle, and 14 of the septal wall of the left ventricle.

Finally, 3747 newborns were included in the analysis, 1635 of which were ≥ 37 weeks. For 2224 and 1551 term and preterm babies, respectively, there were available data ≤ 7DOL, and > DOL7 up to 1 month of life in term infants or up to 44 weeks in preterm infants. An overview of the included studies, the echocardiographic parameters evaluated, and the corresponding JBI quality scores are presented in Supplementary Table 2 (Tab S2). Only thirteen studies were considered “high quality studies” (Tab.S3).

### Pooled estimates of TDI parameters

We conducted meta-analyses for e′, a′, s′ TDI parameter, stratified by gestational age (< 32, 32–36, ≥ 37 weeks) and day of life (≤ 7 or > 7). Summary estimates of pooled means and 95% confidence intervals for each parameter and myocardial wall (RV, LV, IVS) are reported in Tables 1, 2, 3, and 4. Full forest plots for individual studies are presented in the Supplementary Material (Figures S1–S5).

**Table 1:** Normal ranges of s′ tissue Doppler imaging derived parameters in healthy term and preterm newborns

	Number of studies (number of patients)	Mean [95% CI]	Heterogeneity	*p*-value for heterogeneity within time group
Rs′ (cm/s) gestational age ≥ 37 weeks				
* Measurement time* ≤ 7	19 (788)	6.54 [6.09–6.99]	99.5	< 0.001
* Measurement time* > *7*	2 (40)	8.08 [2.95–13.20]	99.6	< 0.001
Gestational age 32–36 weeks				
* Measurement time* ≤ *7*	5 (226)	5.74 [5.52–5.96]	67.5	0.22
* Measurement time* > *7*	1 (48)	9.47 [9.06–9.88]	Not estimable	Not estimable
Gestational age < 32 weeks				
* Measurement time* ≤ *7*	3 (231)	4.91 [4.42–5.39]	94.4	< 0.001
* Measurement time* > *7*	5 (204)	7.63 [6.74–8.51]	96.0	< 0.001
Ls′ (cm/s) gestational age ≥ 37 weeks				
* Measurement time* ≤ *7*	19 (716)	4.90 [4.50–5.29]	99.5	< 0.001
* Measurement time* > *7*	4 (98)	4.71 [3.03–6.38]	98.7	< 0.001
Gestational age 32–36 weeks				
* Measurement time* ≤ *7*	6 (180)	4.47 [4.01–4.92]	92.0	< 0.001
* Measurement time* > *7*	2 (61)	5.71 [5.30–6.12]	48.1	0.17
Gestational age < 32 weeks				
* Measurement time* ≤ *7*	2 (128)	3.92 [3.34–4.50]	94.0	< 0.001
* Measurement time* > *7*	6 (152)	4.92 [4.52–5.32]	91.5	< 0.001
IVSs′ (cm/s) gestational age ≥ 37 weeks				
* Measurement time* ≤ *7*	12 (468)	4.20 [3.79–4.61]	98.0	< 0.001
* Measurement time* > *7*	2 (48)	5.67 [5.38–5.96]	16.4	0.27
Gestational age 32–36 weeks				
* Measurement time* ≤ 7	3 (128)	3.97 [3.52–4.43]	85.9	< 0.001
* Measurement time* > 7	1 (48)	5.54 [5.23–5.85]	Not estimable	Not estimable
Gestational age < 32 weeks				
* Measurement time* ≤ 7	1 (66)	2.90 [2.76–3.04]	Not estimable	Not estimable
* Measurement time* > 7	2 (60)	4.94 [4.72–5.16]	0.0	0.82

*L* left, *R* right, *IVS* interventricular septum

**Table 2:** Normal ranges of e′ tissue Doppler imaging derived parameters in healthy term and preterm newborns

	Number of studies (number of patients)	Mean [95% CI]	Heterogeneity	***p***-value for heterogeneity within time group
Re′ (cm/s) gestational age ≥ 37 weeks				
* Measurement time* ≤ 7	17(595)	7.41 [7.01–7.82]	97.2	< 0.001
* Measurement time* > *7*	2 (40)	12.49 [8.81–16.18]	97.8	< 0.001
Gestational age 32–36 weeks				
* Measurement time* ≤ *7*	3 (87)	5.98 [5.61–6.34]	39.1	0.19
* Measurement time* > *7*	0 (0)	Not estimable	Not estimable	Not estimable
Gestational age < 32 weeks				
* Measurement time* ≤ *7*	2 (152)	4.75 [4.45–5.05]	76.5	0,04
* Measurement time* > *7*	5 (204)	7.92 [6.40–9.44]	96.6	< 0.001
Le′ (cm/s) gestational age ≥ 37 weeks				
* Measurement time* ≤ *7*	19 (705)	6.79 [6.30–7.27]	98.9	< 0.001
* Measurement time* > *7*	5 (145)	8.58 [7.21–9.94]	95.3	< 0.001
Gestational age 32–36 weeks				
* Measurement time* ≤ *7*	5 (132)	6.07 [5.01–7.12]	94.8	< 0.001
* Measurement time* > *7*	1 (13)	5.35 [4.89–5.81]	Not estimable	Not estimable
Gestational age < 32 weeks				
* Measurement time* ≤ *7*	3 (102)	3.78 [3.61–3.95]	0	0.41
* Measurement time* > *7*	6 (152)	4.94 [3.05–6.84]	99.8	< 0.001
IVSe′ (cm/s) gestational age ≥ 37 weeks				
* Measurement time* ≤ *7*	13 (445)	5.27 [4.91–5.64]	92.9	< 0.001
* Measurement time* > *7*	2 (48)	8.56 [7.42–9.70]	69.5	0.07
Gestational age 32–36 weeks				
* Measurement time* ≤ 7	2 (75)	3.51 [1.92–5.10]	98.4	< 0.001
* Measurement time* > 7	0 (0)	Not estimable	Not estimable	Not estimable
Gestational age < 32 weeks				
* Measurement time* ≤ 7	1 (66)	3.23 [3.02–3.44]	Not estimable	Not estimable
* Measurement time* > 7	2 (60)	5.97 [5.55–6.39]	48.7	0.16

*R* right, *L* left, *IVS* intraventricular septum

**Table 3:** Normal ranges of a′ tissue Doppler imaging derived parameters in healthy term and preterm newborns

	Number of studies (number of patients)	Mean [95% CI]	Heterogeneity	*p*-value for heterogeneity within time group
Ra′ (cm/s) gestational age ≥ 37 weeks				
* Measurement time* ≤ 7	16 (574)	9.01 [8.53–9.48]	89.3	< 0.001
* Measurement time* > *7*	1 (20)	13.10 [12.27–13.93]	Not estimable	Not estimable
Gestational age 32–36 weeks				
* Measurement time* ≤ *7*	3 (87)	8.50 [7.52–9.49]	86.4	< 0.001
* Measurement time* > *7*	0 (0)	Not estimable	Not estimable	Not estimable
Gestational age < 32 weeks				
* Measurement time* ≤ *7*	2 (152)	8.29 [8.10–8.48]	0	0.97
* Measurement time* > *7*	5 (204)	11.33 [10.22–12.45]	86.3	< 0.001
La′ (cm/s) gestational age ≥ 37 weeks				
* Measurement time* ≤ *7*	15 (502)	6.54 [6.03–7.04]	91.1	< 0.001
* Measurement time* > *7*	3 (78)	7.55 [6.47–8.63]	74.2	0.02
Gestational age 32–36 weeks				
* Measurement time* ≤ *7*	4 (100)	6.15 [5.16–7.13]	91.9	< 0.001
* Measurement time* > *7*	1 (13)	5.65 [5.24–6.06]	Not estimable	Not estimable
Gestational age < 32 weeks				
* Measurement time* ≤ *7*	3 (102)	4.30 [3.92–4.67]	62.9	0.07
* Measurement time* > *7*	6 (152)	6.61 [3.83–9.40]	99.8	< 0.001
IVSa′ (cm/s) gestational age ≥ 37 weeks				
* Measurement time* ≤ *7*	12 (445)	5.92 [5.23–6.62]	96.7	< 0.001
* Measurement time* > *7*	2 (48)	6.84 [5.96–7.72]	82.6	0.02
Gestational age 32–36 weeks				
* Measurement time* ≤ 7	2 (75)	6.33 [5.97–6.69]	55.7	0.13
* Measurement time* > 7	0 (0)	Not estimable	Not estimable	Not estimable
Gestational age < 32 weeks				
* Measurement time* ≤ 7	1 (66)	4.67 [4.38–4.96]	Not estimable	Not estimable
* Measurement time* > 7	2 (60)	7.52 [7.01–8.03]	0	0.63

*L* left, *R* right, *IVS* interventricular septum

**Table 4:** Normal ranges of cardiac cycle time tissue Doppler imaging derived parameters in healthy term and preterm newborns

	Number of studies (number of patients)	Mean [95% CI]	Heterogeneity	***p***-value for heterogeneity within time group
RV-IVCT (ms) gestational age ≥ 37 weeks				
* Measurement time* ≤ *7*	2 (35)	37.53 [33.87–41.18]	64.5	0.09
* Measurement time* > *7*	0 (0)	Not estimable	Not estimable	Not estimable
Gestational age < 32 weeks				
* Measurement time* ≤ *7*	0 (0)	Not estimable	Not estimable	Not estimable
* Measurement time* > *7*	1 (28)	31.20 [28.19–34.21]	Not estimable	Not estimable
LV-IVCT (ms) gestational age ≥ 37 weeks				
* Measurement time* ≤ *7*	6 (147)	49.18 [42.78–55.58]	97.6	< 0.001
* Measurement time* > *7*	1 (47)	44.49 [42.41–46.57]	Not estimable	Not estimable
Gestational age 32–36 weeks				
* Measurement time* ≤ *7*	2 (59)	49.29 [47.17–51.41]	0.0	0.7
* Measurement time* > *7*	0 (0)	Not estimable	Not estimable	Not estimable
Gestational age < 32 weeks				
* Measurement time* ≤ *7*	2 (92)	45.67 [40.96–50.37]	79.6	0.03
* Measurement time* > *7*	0 (0)	Not estimable	Not estimable	Not estimable
IVS-IVCT (ms) gestational age ≥ 37 weeks				
* Measurement time* ≤ *7*	2 (40)	43.28 [40.61–45.94]	0	0.74
* Measurement time* > *7*	0 (0)	Not estimable	Not estimable	Not estimable
RV-IVRT (ms) gestational age ≥ 37 weeks				
* Measurement time* ≤ *7*	2 (35)	41.53 [40.24–42.81]	0	0.39
* Measurement time* > *7*	0 (0)	Not estimable	Not estimable	Not estimable
Gestational age < 32 weeks				
* Measurement time* ≤ *7*	0 (0)	Not estimable	Not estimable	Not estimable
* Measurement time* > *7*	1 (28)	48.80 [44.50–53.10]	Not estimable	Not estimable
LV-IVRT (ms) gestational age ≥ 37 weeks				
* Measurement time* ≤ 7	5 (122)	46.90 [42.63–51.17]	92.7	< 0.001
* Measurement time* > *7*	2 (77)	41.77 [40.15–43.39]	29.9	0.23
Gestational age 32–36 weeks				
* Measurement time* ≤ 7	1 (32)	44.78 [42.43–47.13]	Not estimable	Not estimable
* Measurement time* > *7*	0 (0)	Not estimable	Not estimable	Not estimable
Gestational age < 32 weeks				
* Measurement time* ≤ 7	1 (66)	54.33 [51.46–57.21]	Not estimable	Not estimable
* Measurement time* > *7*	2 (50)	48.01 [37.82–58.20]	98.9	< 0.001
IVS-IVRT (ms) gestational age ≥ 37 weeks				
* Measurement time* ≤ 7	2 (40)	47.26 [44.37–50.14]	0	0.38
* Measurement time* > *7*	0 (0)	Not estimable	Not estimable	Not estimable
Right filling time (ms) gestational age ≥ 37 weeks				
* Measurement time* ≤ *7*	1 (15)	203.00 [176.52–229.48]	Not estimable	Not estimable
* Measurement time* > *7*	0 (0)	Not estimable	Not estimable	Not estimable
Left filling time (ms) gestational age ≥ 37 weeks				
* Measurement time* ≤ *7*	2 (45)	212.33 [199.27–225.38]	0.0	0.65
* Measurement time* > *7*	0 (0)	Not estimable	Not estimable	Not estimable
Gestational age < 32 weeks				
* Measurement time* ≤ *7*	1 (66)	127.33 [122.44–132.23]	Not estimable	Not estimable
* Measurement time* > *7*	0 (0)	Not estimable	Not estimable	Not estimable
Right ejection time gestational age ≥ 37 weeks				
* Measurement time* ≤ *7*	4 (123)	202.40 [174.51–230.29]	98.3	< 0.001
* Measurement time* > *7*	0 (0)	Not estimable	Not estimable	Not estimable
Gestational age 32–36 weeks				
* Measurement time* ≤ *7*	1 (20)	150.00 [136.85–163.15]	Not estimable	Not estimable
* Measurement time* > *7*	0 (0)	Not estimable	Not estimable	Not estimable
Gestational age < 32 weeks				
* Measurement time* ≤ *7*	1 (38)	191.00 [185.89–196.11]	Not estimable	Not estimable
* Measurement time* > *7*	0 (0)	Not estimable	Not estimable	Not estimable
Left ejection time (ms) gestational age ≥ 37 weeks				
* Measurement time* ≤ *7*	4 (95)	187.84 [179.50–196.19]	80.3	0.002
* Measurement time* > *7*	0 (0)	Not estimable	Not estimable	Not estimable
Gestational age 32–36 weeks				
* Measurement time* ≤ *7*	1 (27)	182.00 [176.11–187.89]	Not estimable	Not estimable
* Measurement time* > *7*	0 (0)	Not estimable	Not estimable	Not estimable
Gestational age < 32 weeks				
* Measurement time* ≤ *7*	2 (92)	153.65 [134.87–172.43]	94.8	< 0.001
* Measurement time* > *7*	0 (0)	Not estimable	Not estimable	Not estimable

*LV* left ventricle, *RV* right ventricle, *IVS* interventricular septum, *IVRT* isovolumetric relaxation time, *IVCT* isovolumetric contraction time

### Certainty of the evidence

Evidence showed a low/very low quality of evidence, according to the GRADE approach (Tab S4). The majority of the studies were downgraded for inconsistency because of heterogeneity, whereas few studies showed a wide confidence interval, and none was downgraded for indirectness considering that we decided to select healthy newborns.

### Data analysis of normal ranges

#### Peak systolic velocity (s′) (Table 1)

Pooled mean values of s′ velocities are summarized in Table 1. A consistent increase in s′ was observed with advancing GA and postnatal age across all myocardial walls. The highest values were reported for the right ventricular wall, particularly after the first week of life, reflecting the rapid postnatal decrease in pulmonary vascular resistance and improved right ventricular contractility. Left ventricular s′ showed a more modest increase with age, likely due to different loading conditions and myocardial architecture. Septal s′ values remained overall lower across all groups. Most studies contributing to s′ estimates were rated as low or very low quality, with considerable heterogeneity.

### Peak early diastolic velocity (e′) (Table 2)

Table 2 reports pooled e′ velocities. Values increased with both GA and postnatal age, consistent with maturing diastolic function. However, in preterm infants, early diastolic velocities remained substantially lower, especially in the septum. Data beyond 7 days were limited for some GA subgroups. Evidence certainty was low to very low, and heterogeneity was high in most estimates.

### Peak atrial diastolic velocity (a′) (Table 3)

As shown in Table 3, a′ velocities increased over time in all myocardial regions, with particularly high values in the right ventricle. As with e′, limited data were available for late postnatal assessments in preterm neonates. The quality of studies was generally low.

### Time intervals (IVRT, IVCT, ET, FT) (Table 4)

Pooled estimates for IVRT, IVCT, ET, and FT are summarized in Table 4. IVRT and IVCT showed considerable variation across GA groups, with generally longer durations observed in preterm infants. Available data were limited, especially for postnatal age > 7 days and for septal measurements. The quality of evidence was low to very low, and pooled estimates were based on few studies per group. These parameters remain promising for research, but their clinical applicability requires further validation.

### S′, e′, and a′ meta-regression analysis

We performed meta-regression on the parameters a′, e′, and s′ as additional explorative analysis. Results are reported in Supplementary Figs. 6–8 (FIG S6-8) and Supplementary Table 5 (Tab S5). We observed significantly positive linear associations with mean GA for all parameters measured before 7 days, with exception of Ra′ and IVSa′, and no association for all parameters measured after 7 days, except for Le′ and IVSs′. However, these associations could be due to chance because of the low number of studies in the meta-regressions.

## Discussion

This is the first meta-analysis to define normal ranges of TDI measurements of myocardial performance in term and preterm neonates, according to GA and postnatal age. We reported reference values for peak systolic and diastolic velocities and cardiac cycle time intervals for both ventricles.

Neonates have faster heart rates than older children and adults, making TDI’s higher temporal resolution advantageous for assessing cardiac function. Unlike conventional echocardiographic techniques (ejection and shortening fraction or pulsed wave Doppler-derived measures), TDI enables comprehensive evaluation of both systolic and diastolic function [3]. However, the lack of robust TDI reference ranges for neonates limits its clinical application. This is partly due to variations in TDI measurements by GA and postnatal age. In early life, dramatic hemodynamic changes occur as the heart transitions from in utero to extra-uterine circulation. Reduced pulmonary resistance and increased left ventricular afterload are major shifts [46, 47], influencing TDI measurements. Additionally, preterm hearts are less compliant and more sensitive to afterload compared to term neonates, with fetal shunts (patent ductus arteriosus (PDA), patent foramen ovale) often remaining patent longer.

Our study accounted for these hemodynamic variations by categorizing populations by GA and DOL.

### Systolic function (s′)

Peak systolic velocity (s′) represents myocardial contraction efficiency, sensitive to preload and afterload changes. Our data show s′ increases in the first DOL across all GA groups, particularly in the right ventricle due to its greater afterload sensitivity. The sharp decrease in pulmonary vascular resistance during the first days after birth leads to a reduction of right ventricular afterload, enhanced contractility, and subsequent increase of pulmonary blood flow. This tendency is less pronounced for the left ventricle, that, due to its myofiber architecture, is less sensitive to the increased afterload in the first days after birth following the removal of the placenta [46, 47].

### Diastolic function (e′ and a′)

TDI’s ability to assess diastolic function is a key advantage. The biphasic diastolic wave consists of early (e′) and late (a′) velocities. In neonates, diastolic function is limited compared to older populations [48], particularly in preterm due to structural and physiological immaturity. Our analysis showed increasing e′ and a′ with both GA and DOL, likely reflecting postnatal hemodynamic changes. We hypothesized that the improvement of left diastolic function in the first days of life may be due to the closure of PDA and the termination of the left volume overload, whereas the reduction of pulmonary vascular resistance leads to an improvement of right ventricular contractility, end diastolic pressure, and atrial pressure.

### Time intervals

TDI-derived time intervals are independent of heart size and angle of insonation, enhancing their comparability across ages. Among them, IVRT is the most studied, correlating with cardiac function [2]. However, its clinical application requires further validation as available data are limited and of low quality.

## Limitations of the study

This meta-analysis has several limitations. First, the study population was divided into three GA groups arbitrarily. While the ranges might seem broad, further subdivisions would have greatly reduced the number of patients in each group, particularly at smaller GAs, preventing the calculation of robust reference ranges. From a practical perspective, this division, alongside the inclusion of only healthy neonates, provides a reasonable starting point for establishing reference ranges.

Secondly, each group was subdivided based on DOL, with a cutoff of 7 days, chosen arbitrarily. Although ideal reference ranges for each DOL would be optimal, data limitations require further studies. Given that the transition from in utero to extra-uterine circulation generally stabilizes within the first week of life, the chosen cutoff was deemed appropriate to reflect cardiac performance without transitional influences.

Thirdly, studies lacking clear GA and DOL information were excluded, possibly omitting high-quality research. However, GA and DOL substantially impact cardiac measurements, and including studies with unclear details would have introduced bias.

Lastly, the high heterogeneity in meta-analyses could be seen as a limitation, but it also reflects the test’s performance across diverse patient groups. For certain TDI parameters like ET, FT, IVRT, and IVCT, insufficient studies were found to establish reliable normal ranges. Nevertheless, these parameters are rarely used in clinical practice.

## Implications for clinical practice

The reference ranges established in this meta-analysis offer clinicians concrete thresholds to interpret TDI values in the neonatal population, stratified by GA and postnatal age. These values provide a structured framework to assess myocardial performance in both term and preterm infants, especially in settings where systolic and diastolic dysfunction is suspected. In clinical practice, these benchmarks may facilitate earlier recognition of abnormal cardiac adaptation, potentially guiding timely interventions. The stratification by age allows for more nuanced interpretation than single-point cutoffs, which are often clinically misleading in neonatology.

## Directions for future research

Future studies should validate these reference ranges in larger, prospective cohorts and in clinical scenarios beyond healthy neonates—such as sepsis, PDA, or perinatal asphyxia. Moreover, there is a need for harmonization of TDI acquisition protocols and for the integration of automated analysis tools to reduce inter-operator variability. Importantly, longitudinal studies should examine whether early TDI deviations predict long-term cardiovascular outcomes, thus defining the prognostic value of these parameters.

## Conclusion

This is the first meta-analysis to define normal ranges of TDI parameters in both term and preterm neonates. This robust set of normative data, stratified by GA and DOL, may enhance the clinical application of TDI, enabling earlier detection of both systolic and diastolic dysfunction, potentially leading to timely interventions and improved outcomes. Further research is needed to refine parameter estimates across sub-populations and to evaluate their clinical utility in improving neonatal outcomes.

## Supplementary Information

Below is the link to the electronic supplementary material.

Supplementary Fig. 1 (Fig S1): Forest plot normal ranges for s’ TDI derived measures (PNG 10.3 MB)

Supplementary Fig. 2 (Fig S2): Forest plot normal ranges for e’ TDI derived measures (PNG 9.95 MB)

Supplementary Fig. 3 (Fig S3): Forest plot normal ranges for a’ TDI derived measures (PNG 9.58 MB)

Supplementary Fig. 4 (Fig S4): Forest plot normal ranges for Isovolumic relaxation (IVRT) and contraction (IVCT) time TDI derived measures (PNG 7.41 MB)

Supplementary Fig. 5 (Fig S5): Forest plot normal ranges for ejection (ET) and filling time (FT) TDI derived measures (PNG 5.99 MB)

Supplementary Fig. 6 (Fig S6): Meta-regression plots for s’ variable (TIF 152 MB)

High Resolution Image (TIF 152 MB)

Supplementary Fig. 7 (Fig S7): Meta-regression plots for e’ variable (PNG 847 KB)

High Resolution Image (TIF 152 MB)

Supplementary Fig. 8 (Fig S8): Meta-regression plots for a’ variable (PNG 447 KB)

Supplementary Table 1 (PDF 28 KB)

## Data Availability

Data are available upon request to the author.

## Abbreviations

NICUs: Neonatal intensive care units
TDI: Tissue Doppler imaging
IVCT: Isovolumic contraction time
IVRT: Isovolumic relaxation time
IVS: Interventricular septum
GA: Gestational age
DOL: Day of life
SD: Standard deviations
CI: Confidence interval
JBI: Joanna Briggs Institute
