# Supplementary figures and images for: Normal ranges of tissue Doppler imaging echocardiographic parameters in healthy term and preterm newborns: a systematic review and meta-analysis
Source: Eur J Pediatr. 2025 Aug 14;184(9):551. doi: 10.1007/s00431-025-06323-1 (PMC12354615; doi:10.1007/s00431-025-06323-1)

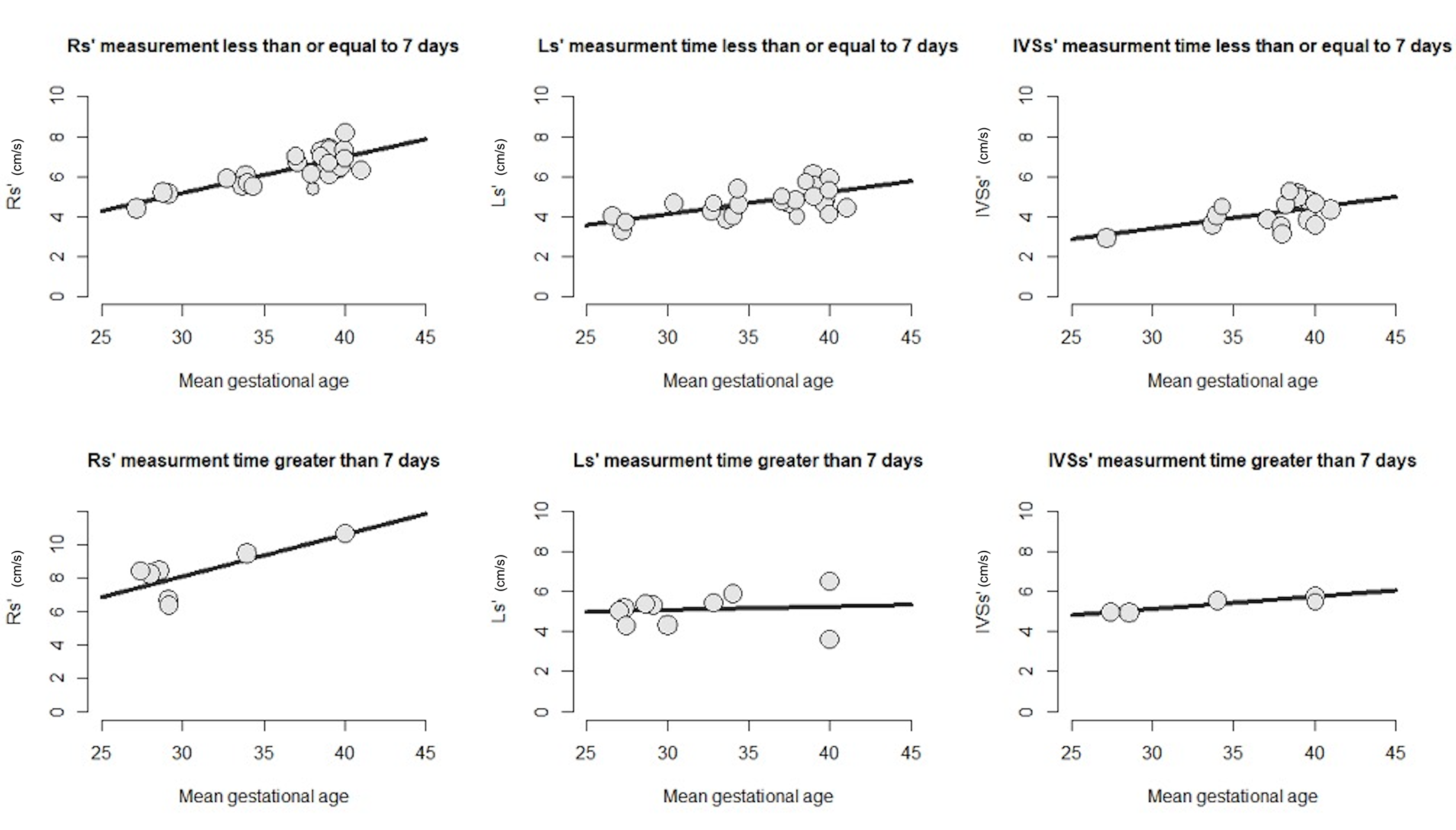

Supplement: Supplementary file 6 — Supplementary Fig. 6 (Fig S6): Meta-regression plots for s’ variable (TIF 152 MB) [file 431_2025_6323_MOESM9_ESM.png]

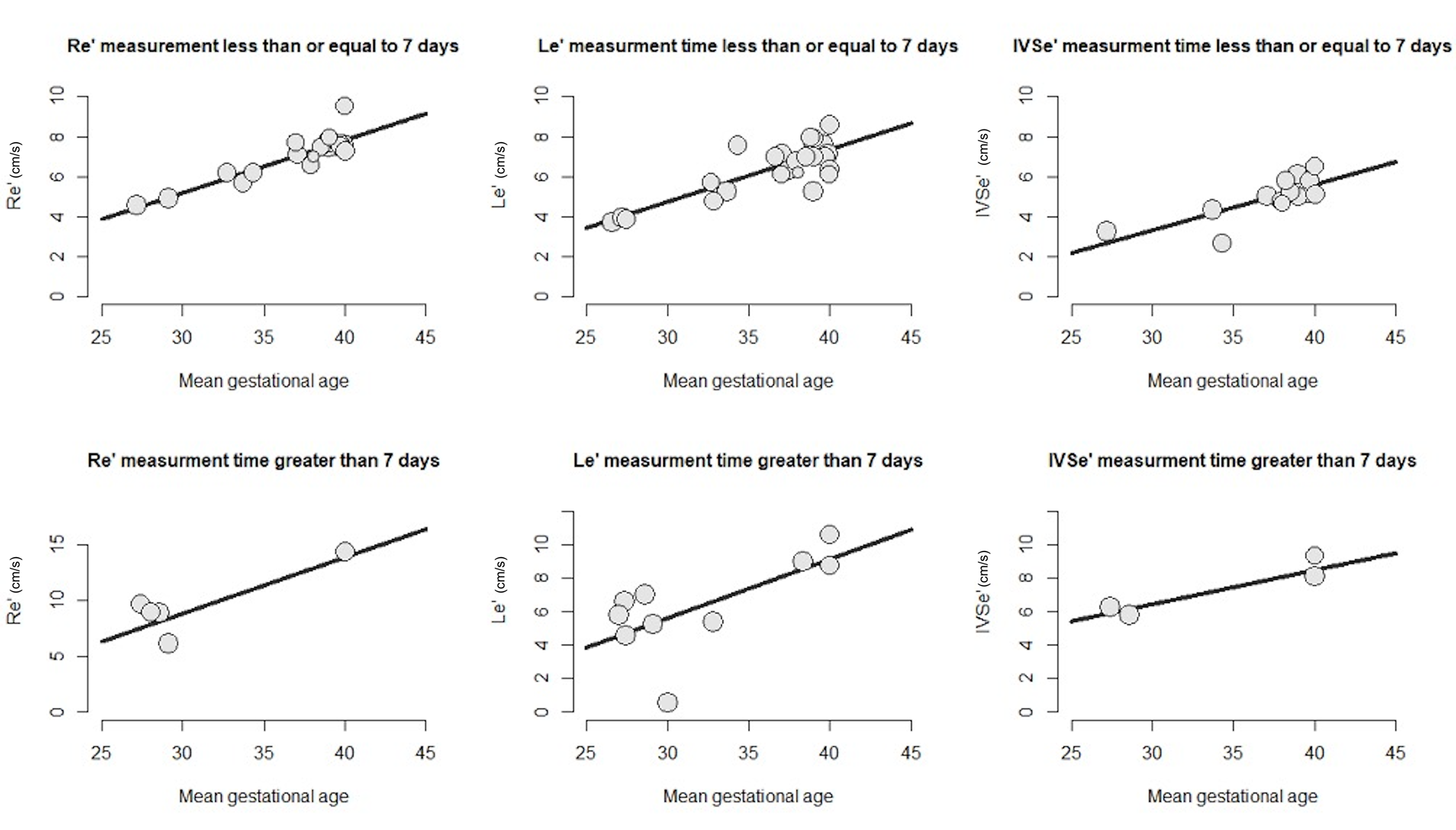

Supplement: Supplementary file 8 — Supplementary Fig. 7 (Fig S7): Meta-regression plots for e’ variable (PNG 847 KB) [file 431_2025_6323_MOESM10_ESM.png]

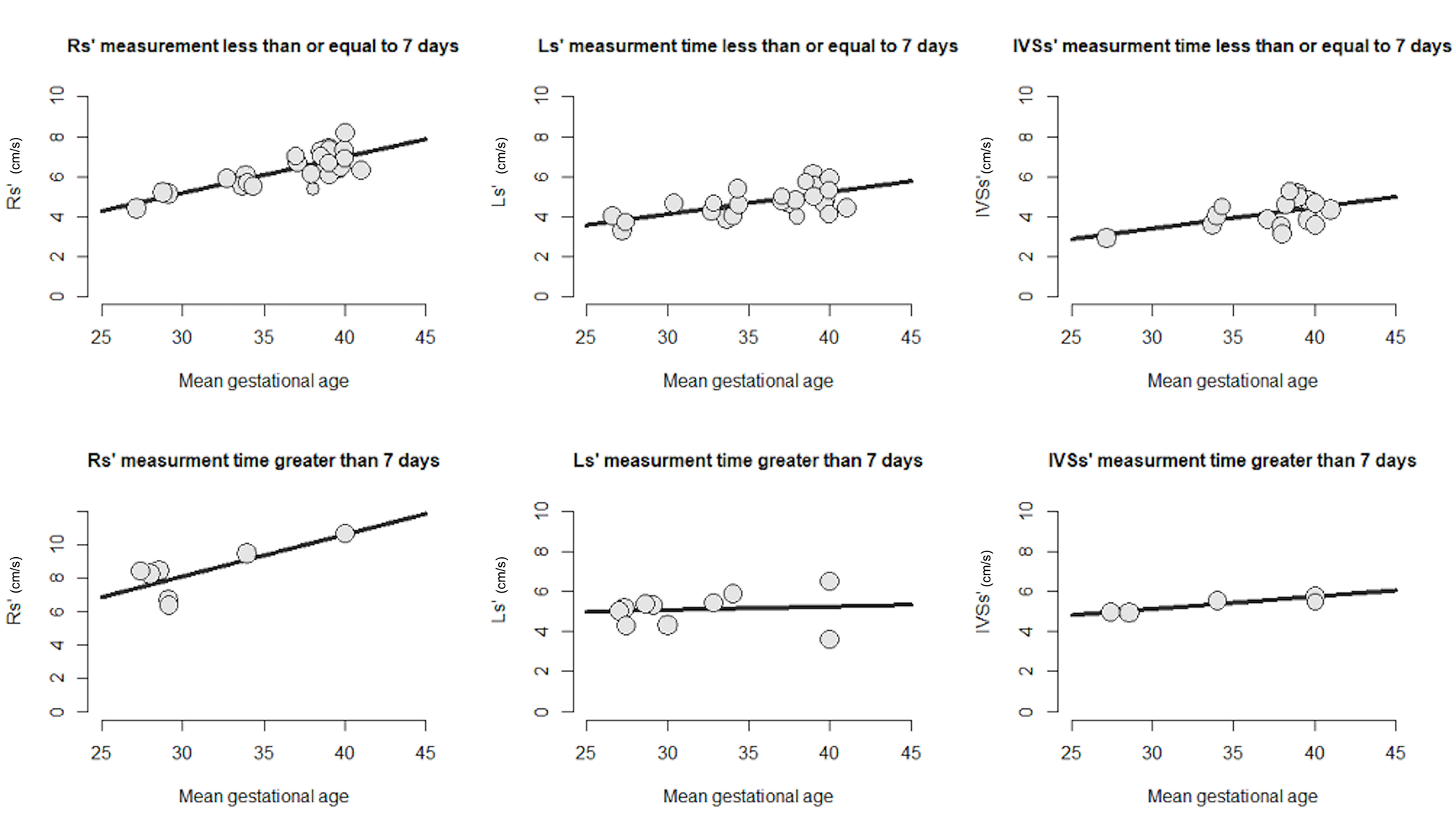

Supplement: Supplementary file 10 — Supplementary Fig. 8 (Fig S8): Meta-regression plots for a’ variable (PNG 447 KB) [file 431_2025_6323_MOESM11_ESM.png]
