# Supplementary material for: Normal ranges of tissue Doppler imaging echocardiographic parameters in healthy term and preterm newborns: a systematic review and meta-analysis
Source: Eur J Pediatr. 2025 Aug 14;184(9):551. doi: 10.1007/s00431-025-06323-1 (PMC12354615; doi:10.1007/s00431-025-06323-1)
Supplement: Supplementary file 12 — Supplementary Table 1 (PDF 28 KB) [file 431_2025_6323_MOESM12_ESM.pdf]

| Database | Search Strategy | Results |
|---|---|---|
| **Embase** | #1: 'newborn'/exp OR 'newborn'<br>#2: newborn* OR neonat* OR infant* OR pediatric OR premature* OR preterm OR 'pre term' OR postmature*<br>#3: #1 OR #2<br>#4: 'echocardiography'<br>#5: 'echography' OR 'diagnostic ultrasound' OR 'ultrasound imaging' OR 'ultrasonic imaging' OR ‘ultrasonographic imaging'<br>#6: #4 OR #5<br>#7: 'tissue doppler imaging' OR (tissue AND doppler) OR tdi<br>Final: #3 AND #6 AND #7 | 936 |
| **PubMed** | #2: Infant, Newborn[MeSH]<br>#3: newborn* OR neonat* OR infant* OR pediatric OR premature* OR preterm OR 'pre term' OR postmature*<br>#4: #2 OR #3<br>#6: Echocardiography[MeSH]<br>#7: ultrasonography OR diagnostic ultrasound OR ultrasound imaging OR ultrasonic imaging OR ultrasonographic imaging<br>#8: tissue doppler OR tissue doppler imaging OR TDI<br>#9: #6 OR #7<br>#10: #8 AND #9<br>Final: #4 AND #10 | 1476 |
| **Cochrane Library** | #1: MeSH descriptor: [Infant, Newborn] explode all trees<br>#2: (newborn* OR neonat* OR infant* OR pediatric OR premature* OR preterm OR 'pre term' OR postmature*)<br>#3: #1 OR #2<br>#4: MeSH descriptor: [Echocardiography] explode all trees<br>#5: echocardiograph*<br>#6: MeSH descriptor: [Ultrasonography] explode all trees<br>#7: ((diagnostic AND imaging) OR ultrasonograph* OR (diagnostic AND ultrasound*) OR (ultrasound* AND imaging) OR (ultrasonic AND imaging) OR (ultrasonographic AND imaging))<br>#8: #4 OR #5 OR #6 OR #7<br>#9: (tissue doppler OR tissue doppler imaging OR TDI)<br>#10: ((tissue* AND doppler AND imag*) OR TDI)<br>#11: #9 OR #10<br>Final: #3 AND #8 AND #11<br>Filters: Language: English, Age: 0-1 month | 132 |
